# Supplementary figures and images for: Effects of functional correction training on injury risk of athletes: a systematic review and meta-analysis
Source: PeerJ. 2021 Mar 25;9:e11089. doi: 10.7717/peerj.11089 (PMC8005292; doi:10.7717/peerj.11089)

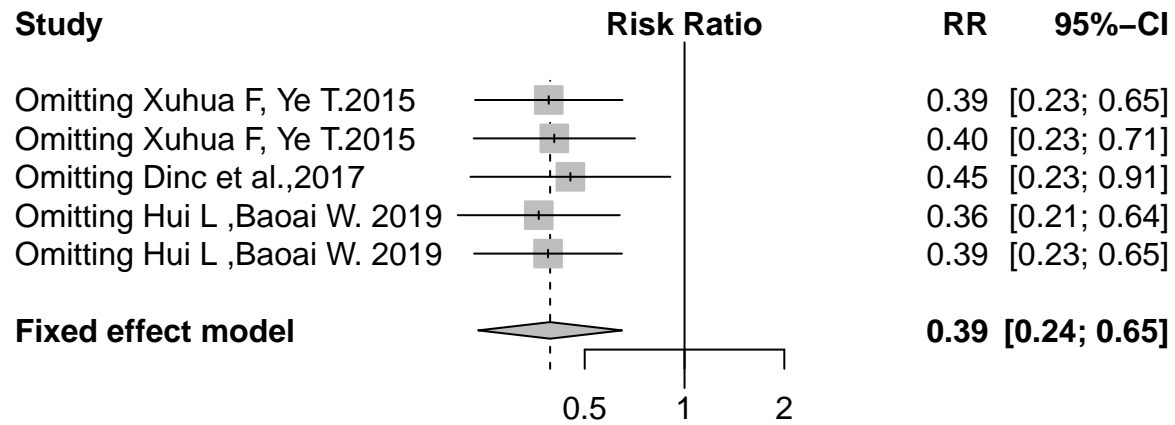

Supplement: Supplemental Information 2 [file peerj-09-11089-s002.pdf]

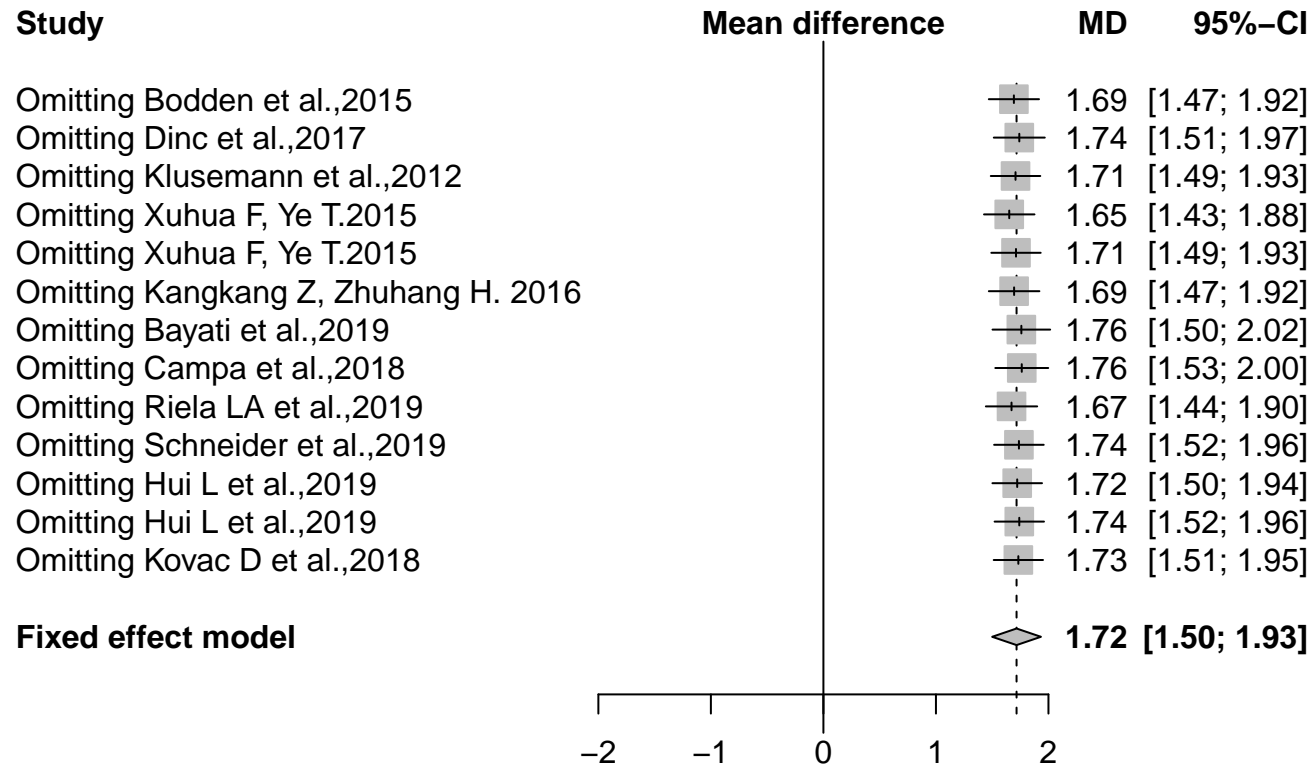

Supplement: Supplemental Information 3 [file peerj-09-11089-s003.pdf]

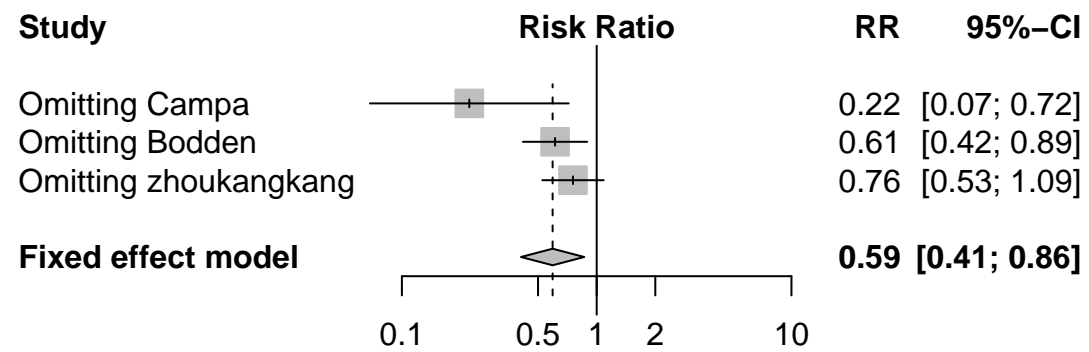

Supplement: Supplemental Information 5 [file peerj-09-11089-s005.pdf]

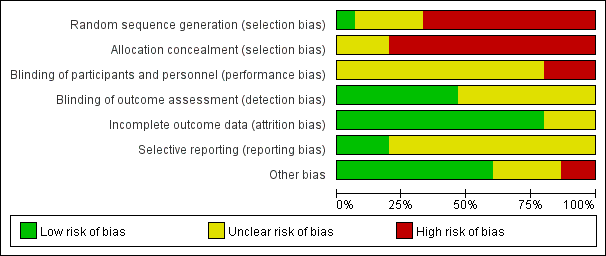

Supplement: Supplemental Information 6 [file peerj-09-11089-s006.png]

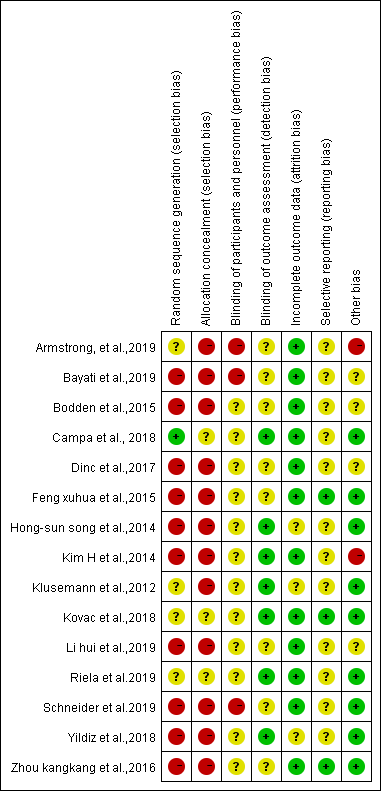

Supplement: Supplemental Information 7 [file peerj-09-11089-s007.png]
