# Supplementary material for: Effects of functional correction training on injury risk of athletes: a systematic review and meta-analysis
Source: PeerJ. 2021 Mar 25;9:e11089. doi: 10.7717/peerj.11089 (PMC8005292; doi:10.7717/peerj.11089)
Supplement: Supplemental Information 4 [file peerj-09-11089-s004.pdf]

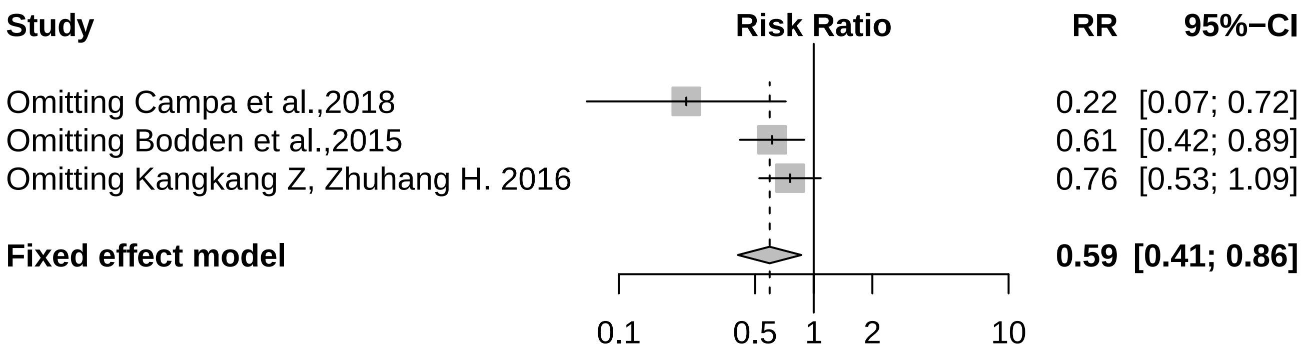
Study
Risk Ratio
RR
95%−CI
Omitting Campa et al.,2018
0.22
[0.07; 0.72]
Omitting Bodden et al.,2015
0.61
[0.42; 0.89]
Omitting Kangkang Z, Zhuhang H. 2016
0.76
[0.53; 1.09]
Fixed effect model
0.59
[0.41; 0.86]
0.1
0.5
1
2
10
