# Supplementary material for: Effects of functional correction training on injury risk of athletes: a systematic review and meta-analysis
Source: PeerJ. 2021 Mar 25;9:e11089. doi: 10.7717/peerj.11089 (PMC8005292; doi:10.7717/peerj.11089)
Supplement: Supplemental Information 8 [file peerj-09-11089-s008.docx]

**Statement of Rationale and Contribution of the work.**

**Effects of Functional Correction Training on Injury Risk of Athletes - A Systematic Review and Meta-analysis**

1. **The rationale for conducting the systematic review / meta-analysis.**

The function of FMS™ was to screen whether the human body's functional patterns were correct, identify potential injury risks, and formulate targeted individual correction plans based on this to improve individual dysfunction movement patterns, thereby may be preventing sports injuries. Past studies focused on predict the sport injury, but the summarized results of systematic reviews and meta-analyses of the total FMS™ score of a mixed population to predict the sports injury risk were contradictory, Whether FMS™ could predict the risk of sports injury, diagnose sports injury was still inconclusive.

To the best of our knowledge, since there is no meta-analysis or systematic review of functional correction training, it is impossible to clearly establish the impact of functional correction training after FMS™ on sports injuries of athletes, needs to be verified through comprehensive evidence.

1. **The contribution that it makes to knowledge in light of previously published related reports, including other meta-analyses and systematic reviews.**

Our results demonstrated the influence of functional correction training based on FMS™ on the sports injury risk of athletes. The injury risk of the experimental group was reduced by 60% after functional correction training, and the effect on the total scores of FMS™ was large, and significantly different from that of the control group. Results of the sensitivity analysis were very robust, and the possibility of publication bias influencing the athletes’ total FMS™ scores was very low. Grade B evidence indicates that functional correction training based on FMS™ could improve athletes’ functional patterns, and Grade D evidence indicates that it could reduce sports injury risks of athletes, The true effect is likely to be different from the estimate of effect, Therefore, further studies are needed to explore the influence of functional correction training on the injury risks of athletes.
